# A Narrative Review of Oxidative Stress and Liver Disease in Pregnancy: The Role of Antioxidants

**DOI:** 10.7759/cureus.64714

**Published:** 2024-07-17

**Authors:** Bandhanjot Kaur, Ravleen K Bakshi, Sujata Siwatch

**Affiliations:** 1 Department of Obstetrics and Gynaecology, Postgraduate Institute of Medical Education and Research, Chandigarh, IND; 2 Department of Health Research, Division of Reproductive Biology, Maternal, and Child Health, Indian Council of Medical Research, Ministry of Health and Family Welfare, New Delhi, IND

**Keywords:** oxidant damage and antioxidants, phytonutrients, antioxidant vitamins, preeclampsia with hellp syndrome, oxidative stress, antioxidants, preeclampsia, liver diseases in pregnancy

## Abstract

Pregnancy brings numerous physiological changes to the body of the pregnant woman. Liver diseases in pregnancy contribute to increased oxidative stress, disrupting the delicate balance between reactive oxygen species and antioxidant defence. Antioxidant supplementation may have potential benefits in addressing pregnancy-related liver disorders, such as HELLP (haemolysis, elevated liver enzymes, low platelet count) and preeclampsia-associated liver dysfunction in pregnancy.

The purpose of this narrative review is to review the evidence regarding oxidative stress in liver disorders during pregnancy and the role of antioxidants in alleviating oxidative stress and its effect on maternal and foetal outcomes. A narrative review study design involved a comprehensive search across three scientific databases: PubMed, Embase, and MEDLINE, published in the last 20 years. The searches were performed up to January 2024.

Thirty-two studies were included in the narrative review. The most studied antioxidants were vitamins (vitamin C and E) for their role in clinical treatment, prophylaxis, and clearing surrogate oxidative stress markers. The majority of studies were on preeclampsia. Though the existing literature is not robust, available evidence suggests that antioxidant supplementation may have potential benefits in addressing pregnancy-related liver disorders, such as HELLP and preeclampsia-associated liver dysfunction in pregnancy. However, there is a need to establish consistent protocols, ethical standards, and well-designed clinical trials to clarify the timing and dosage of antioxidants in pregnancy.

Antioxidants may alleviate the oxidative stress in various liver disorders during pregnancy, which still needs to be studied further for their clinical relevance.

## Introduction and background

Oxidative stress is an imbalance between the oxidant and antioxidant systems in the body. This imbalance leads to a more oxidizing environment in which there is an overproduction of reactive oxygen and nitrogen species [1]. Excessive reactive oxygen species (ROS) production modifies cellular proteins and disrupts the biochemical pathways involved in antioxidant production, causing cellular dysfunction and disruption of vital cellular processes [2,3]. Pregnancy is a physiological state of increased oxidative stress in women. This leads to continuous changes in mitochondrial function, which may include altered gene expression and structure in response to heightened metabolic demands [4,5].

Liver-related disorders in pregnancy pose a health concern not only for the expectant mother but also for the developing foetus. Pregnancy-related liver disorders include pregnancy-related as well as acute and chronic liver disorders that may occur coincidentally during pregnancy. Liver diseases in pregnancy, irrespective of their origin, carry a considerable risk of maternal and foetal morbidity and even mortality [6]. Liver disorders of pregnancy impact 3% of pregnant women, encompassing conditions such as preeclampsia, acute fatty liver of pregnancy, intrahepatic cholestasis of pregnancy (ICP), HELLP (characterized by haemolysis, elevated liver enzymes, and low platelets), and hyperemesis gravidarum [7].

The liver is a crucial organ for metabolism, regulating the body's energy processes. It serves as a critical site for the mitochondrial metabolic pathways that may include adenosine triphosphate (ATP) production, beta-oxidation, the Krebs cycle, and respiratory function, which provide overall energy provision for the body [8]. Thus, liver disorders work to escalate the already heightened oxidative stress in pregnancy.

In this narrative review, we aim to study the liver diseases of pregnancy in relation to the raised oxidative stress and its mechanisms and seek to investigate the potential role of antioxidant supplementation in these diseases. This narrative review intends to provide scientific evidence of the use of antioxidants in treating and improving the adverse outcomes of these diseases.

## Review

Methodology

A comprehensive search was conducted across three scientific databases: PubMed, Embase, and MEDLINE, published in the last 20 years. The searches were performed up to January 2024. The search strategy included various strings using MeSH terms “Pregnant Women” OR "Pregnancy" AND "Antioxidants"[MeSH Terms] OR "Antioxidants"[Pharmacological Action] AND "Supplementation" NOT "animal experimentation" OR "models, animal." The inclusion criteria for the articles were original studies (randomized controlled trials, observational studies, cohort studies, case-control studies) and systematic reviews and meta-analyses in which antioxidants were used as an intervention. The studies focused on only humans. Due to the heterogeneity with respect to the liver disorders, type and dose of antioxidants used, and paucity of published data focusing on the effect of antioxidants on pregnancy outcomes, no quantitative statistical analysis was possible, and the findings are elaborated as a narrative review.

Results and discussion

This narrative review was conducted by reviewing 32 studies related to the use of antioxidants in liver diseases during pregnancy. Key characteristics of the included studies are listed in Table 1.

**Table 1:** Key characteristics of the included studies IUGR: intrauterine growth restriction, UDCA: ursodeoxycholic acid, MDA: malondialdehyde, TBA: total bile acids, ALT: alanine transferase, AST: aspartate transferase, TB: total bilirubin, GSH: glutathione, VAS: visual analog scale, ICP: intrahepatic cholestasis of pregnancy, NAC: N-acetylcysteine, COX-2: cyclooxygenase-2

S. no.	Author	Year of publication	Country	Study design	Sample size	Antioxidants	Main findings
1	Tenorio et al. [9]	2018	Netherland	Systematic review and meta-analysis	Total of 29 studies 19 studies on prevention, 10 studies on treatment	Vitamins C and E, NAC, L-arginine, and resveratrol	No effects in preventing preeclampsia but it showed positive effects in treating IUGR
2	Conde Agudelo et al. [10]	2011	United States of America	Systematic review and meta-analysis	9 trials with 19,810 pregnant women	Vitamins C and E	Did not prevent the risk of preeclampsia during pregnancy, decreased the risk of placental abruption, and increased the risk of premature rupture of membranes and gestational hypertension
3	Polyzos et al. [11]	2007	Italy	Systematic review and meta-analysis	4 trials with 4,680 pregnant women	Vitamin C (1000 mg) and E (400 mg) or placebo per day	Did not reduce the risk of preeclampsia, neonatal loss, small for gestational age infants, or preterm birth
4	Hofmeyr et al. [12]	2018	England	Systematic review and meta-analysis	Total of 27 studies with 18,064 women	High-dose calcium (≥1 g/day) versus placebo: 13 studies (15,730 women), low‐dose calcium (<1 g/day) versus placebo or no treatment: 12 trials (2334 women), high‐dose (=/> 1 g) versus low‐dose (<1 g) calcium: 1 trial (262 women)	High‐dose calcium (≥1 g/day) may reduce the risk for preeclampsia and preterm birth in women with low calcium diets. Increased risk of HELLP with calcium but this was small in numbers. Calcium in low doses did not have a strong impact on stillbirth and preterm birth
5	Gunabalasingam et al. [13]	2023	England	Systematic review and meta-analysis	20 randomized controlled studies	Vitamin C and E calcium vitamin D, folic acid, magnesium, and multiple micronutrients	Reduction in preeclampsia with calcium and vitamin D supplementation
6	Hamdan et al. [14]	2023	Sudan	Systematic review and meta-analysis	Total of 26 studies with the control group: 3,728 healthy pregnant; case group: 855 preeclampsia patients	Selenium	The level of evidence is low. Selenium levels were significantly lower in preeclampsia cases in comparison with control women and women from low- or middle-income countries and the African continent
7	Xu et al. [15]	2016	China	Systematic review and meta-analysis	Total of 16 studies (13 observational studies: 583 preeclampsia patients out of 1515 pregnant women), 3 interventional trials with 218 treated with selenium or placebo out of 439 preeclampsia patients in the third trimester	In interventional studies: selenium 60-100 μg for an average of 5-6 months	Results from observational studies showed that patients with PE have lower levels of Selenium than those compared to healthy pregnant women. Pooled evidence from Interventional trials showed a decrease in the incidence of preeclampsia and an increase in selenium blood levels
8	McDougall et al. [16]	2023	Australia	Systematic review and meta-analysis	Outcome data from 8 trials (1,851 women) and 1 prospective cohort study (71,728 women)	Average selenium dose: 60-200 mg/d	The findings did not determine the effect of selenium supplementation on preterm birth and the risk of perinatal mortality
9	Xu et al. [17]	2022	China	Systematic review and meta-analysis	32 studies (8 trials and 1 cohort study for meta-analysis)	L-arginine	L-arginine supplementation increased plasma nitric oxide concentrations in IUGR pregnancies and elevated birthweights in both hypertensive and IUGR pregnant women. It also reduced the risk of preeclampsia in hypertensive mothers and the risk of small gestational-age foetus significantly
10	Sagadevan et al. [18]	2021	India	Systematic review and meta-analysis	7 studies with preeclampsia patients from 14-32 weeks	L-arginine or placebo or L-arginine and vitamin supplements or placebo	L-arginine showed a significant reduction of preeclampsia and showed no significant effects on maternal gestational age, neonatal weight, and Apgar score
11	Bacq et al. [19]	2012	France	Systematic review and meta-analysis	9 randomized controlled trials, 454 ICP patients in which UDCA: 207, only placebo: 70, cholestyramine: 42, dexamethasone: 36 for 1 week and then placebo for 2 weeks, S-adenosylmethionine: 65, no specific treatment (control group): 34	UDCA, cholestyramine, S-adenosylmethionine, dexamethasone	Pooled analysis compared with controls showed UDCA reduced the pruritus and serum levels of ALT and bile acids. Supplementation with UDCA resulted in reduced foetal distress, fewer premature births, and neonates in the intensive care unit
12	Ovadia et al. [20]	2021	United Kingdom	Systematic review and individual participant data meta-analysis	34 studies (6,974 women with ICP)	UDCA	UDCA had no significant effect on the prevalence of stillbirth in women suffering from ICP
13	Rumbold et al. [21]	2008	United States of America	Systematic review and meta-analysis	10 trials involving 6,533 preeclampsia patients	Combined vitamin C and E therapy	Antioxidant supplementation during pregnancy does not decrease the risk of preeclampsia and other serious complications in pregnancy
14	Mehr and Duley [22]	2010	United Kingdom	Systematic review and meta-analysis	6 studies (310 women)	Nitric oxide donors and precursors	Insufficient evidence to prevent preeclampsia or its complications
15	Patil et al. [23]	2016	India	Prospective intervention study	25 preeclampsia patients in third trimester for 30 days	Vitamin E and Vitamin C	Decreased lipid peroxidation, MDA, triglyceride, and VLDL-C in the treatment group as compared to the start of the treatment
16	Samimi et al. [24]	2016	Iran	Prospective, double-blind, placebo-controlled trial	60 pregnant women (30: intervention group, 30: control group) from 20 to 32 weeks gestation	Vitamin D and calcium supplements or placebo	Increased plasma total GSH concentrations
17	Karamali et al. [25]	2015	Iran	Randomized double-blind placebo-controlled clinical study	60 pregnant women with 20 to 32 weeks gestation	Vitamin D (cholecalciferol) or placebo	Cholecalciferol supplementation significantly increased the plasma total antioxidant capacity concentrations compared with the placebo
18	De Brito et al. [26]	2024	Brazil	Prospective, double-blind, and placebo-controlled study	101 pregnant women with a high risk of preeclampsia from 20 weeks gestation until delivery	Calcium 500 mg calcium/day or 1,500 mg calcium/day or placebo	Improved the inflammatory markers and positive effect on the purinergic system
19	Lorzadeh et al. [27]	2020	Iran	Clinical trial	160 pregnant women without any risk factors for preeclampsia. Case group: vitamin E and vitamin C with iron tablets. Control group: iron tablets for 20-24 weeks of pregnancy	Vitamin E (400 IU/day) and vitamin C (1000 mg/day) with iron tablets and the control group with only iron tablets	Incidence of preeclampsia was higher in the control than in the intervention group. Vitamin C and E have protective effects against preeclampsia by improving the overall blood pressure
20	Roberts et al. [28]	2010	United States of America	Multicenter, randomized, double-blind trial	9,969 nulliparous women with a singleton pregnancy (low risk of preeclampsia) at 9-16 weeks	Vitamins C (1000 mg) and E (400 IU)	Vitamin therapy did not benefit the patients and rates of adverse perinatal outcomes did not differ between the groups
21	Klemmensen et al. [29]	2009	England	Prospective cohort study	57,346 pregnant women	Vitamin C	Low vitamin C dietary intake was associated with an increased incidence of severe preeclampsia, eclampsia, or HELLP. High dietary vitamin E intake increased the incidence of severe disease in a small group of women
22	Antartani et al. [30]	2011	India	Prospective randomized controlled study	54 women with a high risk of developing preeclampsia 14-28 weeks until delivery	Lycopene (4 mg)	Lycopene group had a lesser incidence of growth-restricted babies. It did not decrease the incidence of preeclampsia in high-risk women but had significantly better perinatal outcomes in comparison to the placebo group
23	Sharma et al. [31]	2003	India	Prospective, randomized controlled	251 primigravida women 16-20 weeks until delivery	Lycopene or placebo (4 mg per day)	Lycopene group had a significantly lesser incidence of preeclampsia, average foetal weight was higher, and the occurrence of IUGR was significantly reduced compared to the placebo group
24	Banerjee et al. [32]	2009	India	Prospective randomized double-blind placebo-controlled study	159 primigravida women 12-20 weeks until delivery	Lycopene or placebo (2 mg per day)	Lycopene group had a significantly higher incidence of adverse effects of preterm labour and low birth weight
25	Zhang et al. [33]	2015	China	Multicentered randomized controlled trial	120 pregnant women with ICP. Oral UDCA group: 41, intravenous S-adenosylmethionine: 38, a combination of both drugs: 41	UDCA 250 mg (4 times daily and S-adenosylmethionine (1000 mg daily) until delivery	All therapies significantly and equally improved pruritus. The serum levels of TBA, ALT, AST, and TB significantly decreased in each group after treatment
26	Vadillo-Ortega et al. [34]	2011	Mexico	Randomized controlled trial	672 pregnant women with a high risk of preeclampsia between 14-32 weeks of gestation. Intervention group: 228 women L-arginine plus antioxidant vitamins. Control group: 222 women with antioxidant vitamins alone	L-arginine (6.6 g) and vitamins	The incidence of preeclampsia was reduced significantly
27	Hobson et al. [35]	2018	Australia	Combination of in vitro studies and a phase I clinical trial	20 women with early onset of preeclampsia in the phase I trial	Melatonin (10 mg sustained-release formulation, Natrol, Chatsworth, CA). 1 tablet (3 times a day) daily from recruitment until delivery	Melatonin therapy extends the duration of pregnancy and reduces the need for increasing antihypertensive medication in women with severe preeclampsia
28	Roes et al. [36]	2006	Netherlands	Randomized, double-blind, placebo-controlled trial	38 women with preeclampsia out of 59 women 25-33 weeks until delivery	NAC or placebo: 600 mg per day	No significant difference in the NAC and placebo groups in terms of the median treatment-to-delivery interval and plasma homocysteine concentrations. It did not stabilize severe preeclampsia or HELLP syndrome
29	Motawei et al. [37]	2016	Egypt	Case-control	125 (pregnant women with preeclampsia treatment group: 50; comparator group: 50; control group: 25 pregnant women without preeclampsia)	NAC: 400 mg per day or conventional treatment	Improved APGAR scores and birth weight with NAC. No difference was observed in the oxidative stress markers between the groups
30	Aalami-Harandi et al. [38]	2015	Iran	Randomized, double-blind, placebo-controlled trials	44 pregnant primigravida women at 27 weeks of gestation	Garlic tablet (equal to 400 mg garlic and 1 mg allicin) once daily for 9 weeks	Decreased levels of serum high-sensitivity C-reactive protein and increased plasma GSH
31	Fadinie et al. [39]	2020	Indonesia	Double-blind randomized design	46 preeclampsia patients (case group: 23 and control group: 23)	Curcumin (100 mg)	Significantly decreased COX-2 level, decreased VAS values, shorter clotting time, and lower thrombocyte count
32	Miraj and Baghbahadorani [40]	2016	Iran	Randomized controlled trial	60 patients whose pregnancy ended from severe preeclampsia (case group: 30, control group: 30)	Silymarin (70 mg) after the termination of the pregnancy	No significant differences in lipid peroxidation between the silymarin-treated group and the control group

Oxidative stress plays a crucial role in the pathogenesis of liver diseases during pregnancy. Oxidative stress, i.e., the imbalance of oxidants and the body's antioxidant defence system, disrupts normal cellular function. ROS may include free radicals and reactive molecules [41]. Endogenous sources may include cell organelles like mitochondria, peroxisomes, and endoplasmic reticulum that consume oxygen in many metabolic pathways and produce ROS. Exogenous factors like environmental pollutants, ionizing and non-ionizing radiations, smoking, alcohol, drugs, etc. induce oxidative stress and reduce the antioxidant capacity of the body [42]. Oxidative stress in the liver is produced by endogenous and exogenous sources.

Mechanism of Oxidative Stress in Liver Diseases in Pregnancy

Acute fatty liver of pregnancy (AFLP): AFLP is a rare and severe condition associated with high rates of maternal and foetal morbidity and mortality. Mitochondrial dysfunction plays an essential role in the development of ROS, which leads to inflammation. There is a strong association between maternal AFLP and disrupted mitochondrial fatty acid β-oxidation in the foetus and placenta; specifically, several reports on long-chain hydroxyacyl-CoA dehydrogenase (LCHAD) deficiency have been reported. LCHAD is a component of the mitochondrial trifunctional protein and is required for fatty acid metabolism. Mitochondrial β-oxidation involves four enzymatic reactions. Deficiency of the third enzyme, LCHAD, leads to the accumulation of medium- to long-chain fatty acids in maternal blood and hepatocytes. This can cause toxic effects on maternal hepatocytes such as microvesicular steatosis, disruption of β-oxidation, decreased ATP production, and oxidative stress [43].

Preeclampsia and HELLP: Preeclampsia and HELLP patients are associated with oxidative stress and nitrative stress in both the maternal bloodstream and the placenta. The placental barrier comprises placental trophoblasts and endothelial cells, which separate foetal and maternal circulation. Preeclampsia is characterized by poor trophoblast invasion and poor spiral artery remodelling that leads to placental oxidative stress and nitrative stress with generalized endothelial dysfunction. In patients with preeclampsia, oxidative stress disrupts the integrity of the placental barrier, leading to the transfer of placenta and foetal-derived factors into the maternal circulation. This influx of placenta-derived factors contributes to endothelial damage in the mother, raises oxidative stress, and triggers systemic inflammation [44].

ICP: ICP is characterized by skin itching, an increase in serum alanine aminotransferase levels, and a total bile acid concentration in the mother that has been linked with the risk of foetal loss. Raised bile acids promote the generation of ROS, which subsequently release aminotransferases, alkaline phosphatases, bilirubin, and γ-glutamyl transpeptidases into the bloodstream [45].

Hyperemesis gravidarum: Hyperemesis gravidarum is another gestational disorder marked by intense and persistent nausea and vomiting that extends beyond the first trimester. This leads to exhaustion of maternal nutrient stores, leading to weight loss (>5%), dehydration, ketonuria, ketonemia, and electrolyte and acid-base imbalance. There is heightened lipid peroxidation and an increase in certain inflammatory markers [46].

Antioxidants

As per the National Cancer Institute, "antioxidants are the substances that protect cells from the damage caused by free radicals (unstable molecules made by the process of oxidation during the normal metabolism)," and Halliwell and Gutteridge defined antioxidants as "any substance that delays, prevents, or removes oxidative damage to a target molecule" [47]. Antioxidants are used for many therapeutic purposes. Antioxidants neutralize ROS and mitigate oxidative stress, which improves maternal-foetal outcomes in pregnant women with liver-related diseases. The classification of the antioxidants that are most commonly used in clinical settings is described in Table 2.

**Table 2:** Common antioxidants and their proposed benefits in various liver diseases NAFLD: non-alcoholic fatty liver disease, ALT: alanine transferase, NASH: non-alcoholic steatohepatitis, AST: aspartate transferase, HDL: high0density lipoprotein, TNF: tumor necrosis factor, BMI: body mass index, MDA: malondialdehyde, hs-CRP: high-sensitivity C-reactive protein, TBARS: thiobarbituric acid reactive substances, TAC: total antioxidant capacity, ALP: alanine phosphatase, HDL-C: high-density lipoprotein cholesterol, IL: interleukin, NAC: N-acetylcysteine

Antioxidants	Average dose	Used in diseases	Proposed/anticipated benefits	References
Non-enzymatic/secondary				
Glutathione	300 mg/day	NAFLD	Decreased ALT, triglycerides, and ferritin levels	[48]
Alpha lipoic acid	300 mg/day	NASH, NAFLD	Decreased ALT and AST levels, increased HDL, reduced TNF alpha, triglyceride levels	[49]
NAC	100-150 mg/kg body weight	Acute liver failure	Improved mitochondrial functions and reduced oxidative stress	[50]
Water soluble				
Vitamin C	500-1000 mg/day	Hepatitis	Inhibited lipid peroxidation and reduced Liver function enzymes	[51]
Vitamin E	200-900 IU/day	NAFLD	Decreased AST, ALT, and BMI	[52]
Fat-soluble				
Ergothioneine	5 to 25 mg/day	Healthy humans	Decreased oxidative damage	[53]
Vitamin D	50,000 IU/day	NAFLD	Decreased serum MDA, hs-CRP	[54]
Zinc	30 mg/day	NAFLD	Decreased ALT, γ-glutamyl transpeptidase enzymes	[55]
Molecular Hydrogen	330 mL water thrice a day with tablets producing hydrogen-rich water (>4 mg/L H_2_)	NAFLD	Increased the efficiency of oxidative phosphorylation, coenzyme q10, decreased TBARS	[56]
Plant-derived				
Silymarin	420-600 mg/day	ALD, NAFLD, NASH, Hepatitis	Reduced oxidative stress and hepatic dysfunction	[57]
Green coffee extract	200 mg twice a day	NAFLD	Reduced systolic blood pressure and body mass index	[58]
Sour tea	450 mg /day	NAFLD	Decreased serum triglycerides, ALT, AST, systolic and diastolic blood pressure, and increased TAC levels	[59]
Sativa oil	20 g/day	NAFLD	Improved inflammation and metabolic endotoxemia	[60]
Ginger powder	500 mg/day	NAFLD	Decreased ALT, hs-CRP, and total cholesterol	[61]
Curcumin	1500 mg/day	NAFLD	Decreased hepatic fibrosis, and nuclear factor kappa B activity	[62]
Piperine	5 mg/day	NAFLD	Reduced hepatic enzymes, insulin resistance, and improved lipid and glucose metabolism	[63]
Turmeric	3 g/day	NAFLD	Decreased serum ALP and increased HDL-C	[64]
Mastiha	2 g/day	NAFLD	Improved total antioxidant status levels	[65]
Bayberry juice	250 ml/day	NAFLD	Decreased plasma levels of carbonyl groups, TNF alpha, IL-8	[66]
Dark chocolate	30 g/day	NAFLD	Decreased AST levels	[67]
Glycyrrhizin	200 mg/day	Hepatitis B	Decreased ALT levels	[68]
Phyllanthus niruri	1000 mg/day	Alcoholic hepatitis	Increased total antioxidant status	[69]

Key Antioxidants for Liver Disease in Pregnancy

Vitamins: Vitamin C and E are widely studied to improve the outcome of preeclampsia and HELLP patients. In the study by Lorzadeh et al., vitamins C and E were shown to have protective effects against preeclampsia [27]. A systematic review and meta-analysis was done on oral antioxidants in 2018 by Tenorio et al., comprising 19 randomized controlled studies. The maternal study outcomes were the rate of preeclampsia and perinatal death, while the foetal outcomes were intrauterine growth restriction, low birth weight, premature death, miscarriage, small for gestational age, and incidence of admission to the neonatal intensive care unit. Pooled risk ratios showed no beneficial effects of antioxidants. L-arginine showed a beneficial effect on reducing intrauterine growth restriction [9]. Patil et al.'s study showed that administration of vitamins E and C resulted in a significant decrease in lipid peroxidation (MDA), triglyceride levels, and very low-density lipoprotein cholesterol on the 15th and 30th days of vitamin treatment as compared to baseline (Day 0), with a p-value less than 0.001 [23]. In contrast, Conde-Agudelo et al.'s study resulted in an increased risk of premature rupture of membranes and gestational hypertension in women supplemented with vitamins C and E, while the risk for abruptio placentae was decreased. However, no significant difference in the risk of adverse maternal or foetal/perinatal outcomes was observed between the two groups [10]. The results were consistent with a previous study by Polyzos et al. [11]. Roberts et al. showed that vitamin treatment did not result in significant changes in rates of preeclampsia, severe preeclampsia, or HELLP syndrome [28]. However, in a study by Klemmensen et al., a low intake of vitamin C in the diet showed a trend towards an increased frequency of severe preeclampsia, eclampsia, or HELLP, while a high intake of vitamin E from supplements and dietary sources resulted in a small increase in severe preeclampsia [29]. Rumbold et al.’s Cochrane systematic review investigated the impact of multivitamins on the risk of preeclampsia. Results were uncertain due to inconsistencies and high statistical heterogeneity [21]. Samimi et al. studied women who were at risk for preeclampsia. Administration of vitamin D along with calcium for 12 weeks had positive effects on blood sugar levels, high-density lipoprotein cholesterol, glutathione, and blood pressure. [24] Similarly, Karamali et al. found that cholecalciferol (vitamin D) supplementation for 12 weeks had favourable effects on insulin function measures, serum HDL cholesterol, and total antioxidant capacity concentrations [25].

Calcium: Calcium is one of the macrominerals required by the body for adequate growth, bone development, and nerve function and is administered during pregnancy. It also functions as a scavenger for free radicals. De Brito et al. studied the consequences of supplementing calcium on oxidative stress in pregnant women who are at risk for preeclampsia. The administration of calcium for six weeks exhibits antioxidant action and positively influences the purine signalling pathway and inflammatory markers [26]. The Cochrane review by Hofmeyr et al. showed that women with a low calcium diet if supplemented with a high dose of calcium, i.e., more than equal to 1 g/day, may reduce the risk of preeclampsia and preterm birth. However, the quality of the evidence was low, with publication bias. An increased risk of HELLP syndrome was also noted in a small number with calcium supplementation [12]. In 2023, a systematic review published by Gunabalasingam et al. studied the impact of micronutrients, i.e., vitamins C, E, and D, calcium, folic acid, and magnesium, as interventions for preeclampsia. The results from this study showed a lower incidence of preeclampsia associated with calcium and vitamin D. However, the overall effect of micronutrients on the occurrence of severe preeclampsia was not significant [13].

Selenium: Selenium is one of the trace elements needed in small quantities, i.e., below 100 mg; more than that can be toxic to humans. It also functions as an antioxidant. Hamdan et al., in their meta-analysis, suggested that selenium can be used as an antioxidant for prophylaxis in pregnant women who are at risk of preeclampsia [14]. Xu et al.'s study also stated that selenium significantly reduces the incidence of preeclampsia [15]. McDougall et al. assessed the effect of selenium supplementation on maternal and foetal outcomes, but no significant association was found between the effect of selenium supplementation and preterm birth at less than 37 and 34 weeks of gestation [16]. Overall, limited evidence supports selenium supplementation during pregnancy or for liver diseases in pregnancy.

Lycopene: Lycopene is a lipophilic carotenoid found in red fruits and vegetables with anti-inflammatory, antioxidant, and antihypertensive properties. In a study by Antartani et al., lycopene supplementation appears to be protective in preeclampsia patients. The supplemented group exhibited significantly improved perinatal outcomes [30]. In another study by Sharma et al., preeclampsia occurred significantly less frequently in the lycopene group compared to the placebo group. Additionally, when comparing the lycopene group with the placebo group, intrauterine growth restriction was significantly lower [31]. In contrast, Banerjee et al.'s study showed that there was no benefit of lycopene in the prevention of preeclampsia, and in their study, a higher incidence of adverse effects like preterm labour and low birthweight were noted [32].

Nitric oxide and its precursors: L-arginine is the precursor of nitric oxide that functions as a vasodilator, inhibits platelet aggregation, and limits thrombus formation. Xu et al. studied the supplementation of arginine (a precursor of nitric oxide) in preeclampsia. Results showed that l-arginine supplementation decreased the risk of preeclampsia in mothers with hypertensive disorders. Moreover, administering L-arginine during pregnancy significantly lowered the risk of infants being small for gestational age in pregnancies affected by hypertensive disorders and intrauterine growth restriction [17]. A systematic review and meta-analysis by Sagadevan et al. reported that L-arginine demonstrated a significant decrease in preeclampsia. However, it did not have significant effects on maternal and neonatal outcomes [18]. In Vadillo-Ortega et al.'s study, preeclampsia was reduced significantly in the L-arginine plus antioxidant vitamins group when compared with placebo [34]. A Cochrane review by Meher and Duley did not draw consistent conclusions concerning the effects of these interventions on preeclampsia as the data available was insufficient [22].

Melatonin: Melatonin is known for its antioxidant, anti-inflammatory, and vascular protective properties. It has been observed that in cases of severe preeclampsia, there is a notable decrease in circulating melatonin levels and placental melatonin receptor expression compared to a normal pregnancy. Hobson et al. conducted a study on the placental explant model in a small group of pre-eclamptic women. The results indicated that melatonin does not influence the production of anti-angiogenic factors (sFlt, sEng, and activin A) in placental explants. Additionally, in the phase 1 trial, melatonin did not demonstrate any improvement in either oxidative stress markers or endothelial dysfunction. However, it notably extended the interval between diagnosis and delivery by six days when compared with historical controls. These findings may suggest that melatonin has the potential to enhance perinatal outcomes and reduce morbidity and mortality among preeclampsia patients [35].

N-acetylcysteine (NAC): glutathione is the antioxidant implicated in preeclampsia and HELLP syndrome pathophysiology. NAC has been utilized as a precursor in glutathione synthesis and functions as a direct scavenger for ROS. In a double-blind, placebo-controlled study by Roes et al., it was found that in the control group, plasma glutathione levels 24 hours after delivery decreased among the placebo group, while those who received NAC exhibited increased plasma cysteine concentrations six weeks postpartum. Oral NAC administration did not show any visible benefits in stabilizing HELLP syndrome [36]. In a study by Motawei et al. on preeclampsia patients, NAC improved liver and kidney functions. NAC supplementation decreased the blood pressure and reduced the levels of proteinuria. Improved birth weight and Apgar scores of newborns were also observed in the patients treated with NAC [37]. In treating acute fatty liver during pregnancy, current European Association for the Study of Liver (EASL) guidelines do not confirm or deny the effectiveness of N-acetylcysteine. Nonetheless, its positive effects have been observed in managing liver failure that is not related to acetaminophen overdose. NAC is currently recommended in all patients with acute liver failure, irrespective of etiology [50,70]. Therefore, it may be an option for pregnant women with severe AFLP who require ICU admission [71].

Ursodeoxycholic acid (UDCA): UDCA is recommended as the only first-line treatment for ICP in various national guidelines. It is the only FDA-approved drug for managing ICP. UDCA has been extensively studied for ICP [19-20,33]. Other than UDCA, antioxidants like S-adenosyl-L-methionine, glutathione, silymarin, and coenzyme Q have been studied for their therapeutic potential as antioxidants and anti-inflammatory properties [72]. According to the EASL guidelines for the management of liver diseases, UDCA is safe during pregnancy and breastfeeding. There is a strong recommendation and strong consensus on its use in ICP.

Phytonutrients: Phytonutrients, also known as phytochemicals, are the compounds found in plants that have certain antioxidant and anti-inflammatory properties. Garlic, or *Allium sativum L*, is also known for its antioxidant properties. In a study by Aalami-Harandi et al. on pregnant women at risk of preeclampsia, the administration of garlic reduced the highly reactive C-reactive protein levels and increased plasma glutathione levels. No notable impact of garlic consumption on levels of serum lipid profiles and plasma total antioxidant capacity was identified [38]. Curcumin is the active substance of turmeric or curcumin longa. It is a widely used spice in Indian cuisine. Curcumin has anti-platelet properties by inhibiting the activity of cyclooxygenase-2 (COX-2), an enzyme crucial in inflammation in preeclampsia cases. Fadinie et al. studied that in preeclampsia patients, COX-2 in the placenta is found to be increased, contributing to increased thromboxane production. More thromboxane and less prostacyclin are produced in the placentas of preeclamptic patients than in normal pregnant females. It was observed that even a single 100-mg dose of curcumin was able to reduce the level of COX-2 serum administered 12 hours post-surgery. Curcumin significantly decreased the mean COX-2 levels between the cases and control groups (p=0.001), thus preventing the progression of preeclampsia to eclampsia [39]. Silymarin is a medicinal plant with antioxidant properties. A double-blind interventional study was conducted by Miraj and Baghbahadorani on patients with severe preeclampsia. After pregnancy termination, the intervention group showed a statistically significant higher mean antioxidant capacity in maternal serum (1.4±0.23) compared to the control group (0.9±0.18) at the 60-hour mark. In terms of lipid peroxidation, no significant difference was observed in maternal serum between the two groups [40].

Limitations

The utilization of antioxidants as a potential intervention for liver diseases in pregnancy faces several challenges, as every pregnancy is unique and complex. Though oxidative stress plays a crucial role in the causation of preeclampsia and liver disease in pregnancy, the role of antioxidants in the prevention of liver disease and preeclampsia is not definitively proven. There is a need to perform large multicentric international studies on the role of antioxidants before recommending their routine use in clinical practice. The variations in study groups, such as location, ethnicity, and socioeconomic status, present in the studies conducted on antioxidants add to the challenge of interpreting results uniformly. The absence of standardized protocols for antioxidant use, including differences in doses, types, and timing, makes direct comparisons of studies difficult. Determining the optimal timing for administering antioxidants during pregnancy to maximize their benefits adds to the challenge. Furthermore, safety concerns regarding antioxidant supplements, especially at higher doses, need thorough evaluation, particularly during crucial stages of foetal development and potential interactions with other medications or nutrients. This raises concerns about safety and the possibility of negative outcomes.

## Conclusions

This review examined the role of antioxidants in the prevention and management of liver diseases during pregnancy. The findings suggest that antioxidant supplementation may offer potential benefits in addressing pregnancy-related liver disorders, such as HELLP, and preeclampsia-associated liver dysfunction, though there may be individual differences in oxidative stress and antioxidant needs during pregnancy. While many antioxidants show theoretical promise for reducing oxidative stress during pregnancy in animal models, translating these findings into clinical practice remains unexplored. Efforts to determine the safety, effectiveness, and best practices for using antioxidants in diverse groups of pregnant women are essential. Ongoing research on antioxidants reveals numerous molecules that have the potential for clinical use. However, there is a need for definite evidence and longitudinal studies to track maternal and foetal outcomes after antioxidant supplementation. Collaborations among researchers and conclusive evidence to guide the optimal use of antioxidants are the need of the hour to fully harness the potential of antioxidants in liver-related diseases in pregnancy.
